# Author Correction: Muscle pathology from stochastic low level DUX4 expression in an FSHD mouse model

**DOI:** 10.1038/s41467-018-03449-9

**Published:** 2018-02-22

**Authors:** Darko Bosnakovski, Sunny S. K. Chan, Olivia O. Recht, Lynn M. Hartweck, Collin J. Gustafson, Laura L. Athman, Dawn A. Lowe, Michael Kyba

**Affiliations:** 1 Lillehei Heart Institute, University of Minnesota, Minneapolis, MN 55455 USA; 2 Department of Pediatrics, University of Minnesota, Minneapolis, MN 55455 USA; 3 Faculty of Medical Sciences, University Goce Delcev-Stip, Stip, 2000 Macedonia; 4 Division of Rehabilitation Science and Division of Physical Therapy, Department of Rehabilitation Medicine, University of Minnesota, Minneapolis, MN 55455 USA

Correction to: *Nature Communications* 10.1038/s41467-017-00730-1, published online 15 September 2017

In the originally published version of this Article, an incorrect grant number, RO1 NS083549, was acknowledged. The correct grant number is RO1 AR055685. This error has now been corrected in both the PDF and HTML versions of the Article.

