# Supplementary material for: Muscle pathology from stochastic low level DUX4 expression in an FSHD mouse model
Source: Nat Commun. 2017 Sep 15;8:550. doi: 10.1038/s41467-017-00730-1 (PMC5601940; doi:10.1038/s41467-017-00730-1)
Supplement: Supplementary file 1 — Supplementary Information Supplementary Figures and Supplementary Tables [file 41467_2017_730_MOESM1_ESM.pdf]

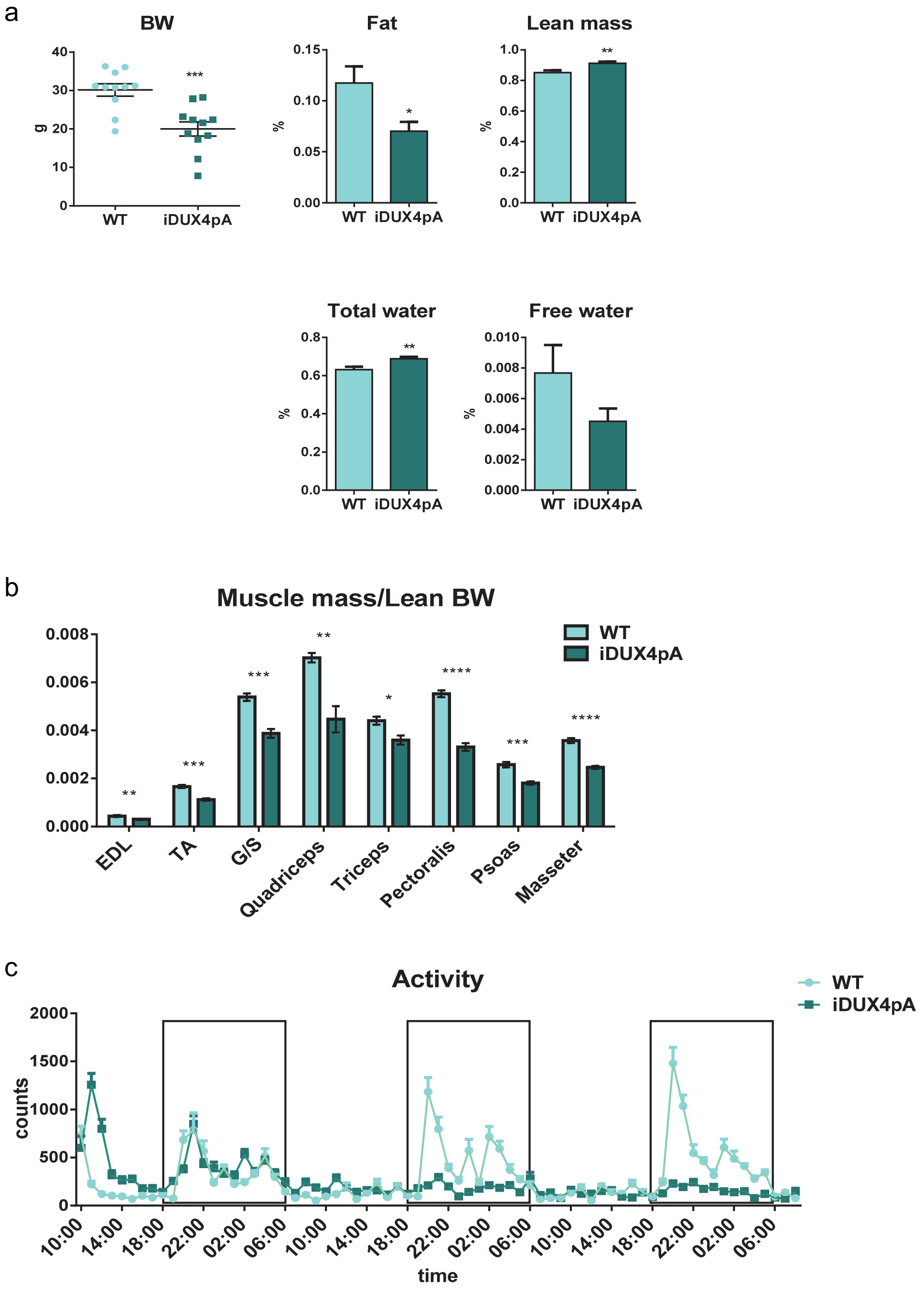

Supplementary Figure 1. Fat and water content, lean body mass-normalized muscle weight, and activity.

(a) Body weight (BW), percent fat mass, percent of fat free mass, percent free water and percent total water in 4 to 12 week old iDUXpA males and WT siblings (n=12). (b) Mass normalized to lean body weight (as opposed to data in Fig. 1 which is normalized to total body weight) of various muscles from 10 to 12 week old iDUX4pA males and WT siblings (n=5). Because of the paucity of fat in the iDUX4pA mice, the difference in total BW between WT and iDUX4pA is larger than the difference in lean BW, therefore the relative loss of muscle mass is larger and of greater significance when normalizing to lean BW. (c) Hourly average locomotor activity of iDUXpA males and WT siblings measured in calorimetry chambers over 72 hours. Boxed areas indicate 12 hour dark periods, in which mice are expected to be more active. Note that iDUX4pA mice are more active immediately after entry into the new environment on day 1, but less active thereafter (n=6). Data are presented as mean +/- SEM; *p<0.05, **p<0.01, ***p<0.001, ****p<0.0001 by T-test or (c) two-way ANOVA with Sidak's post hoc test.

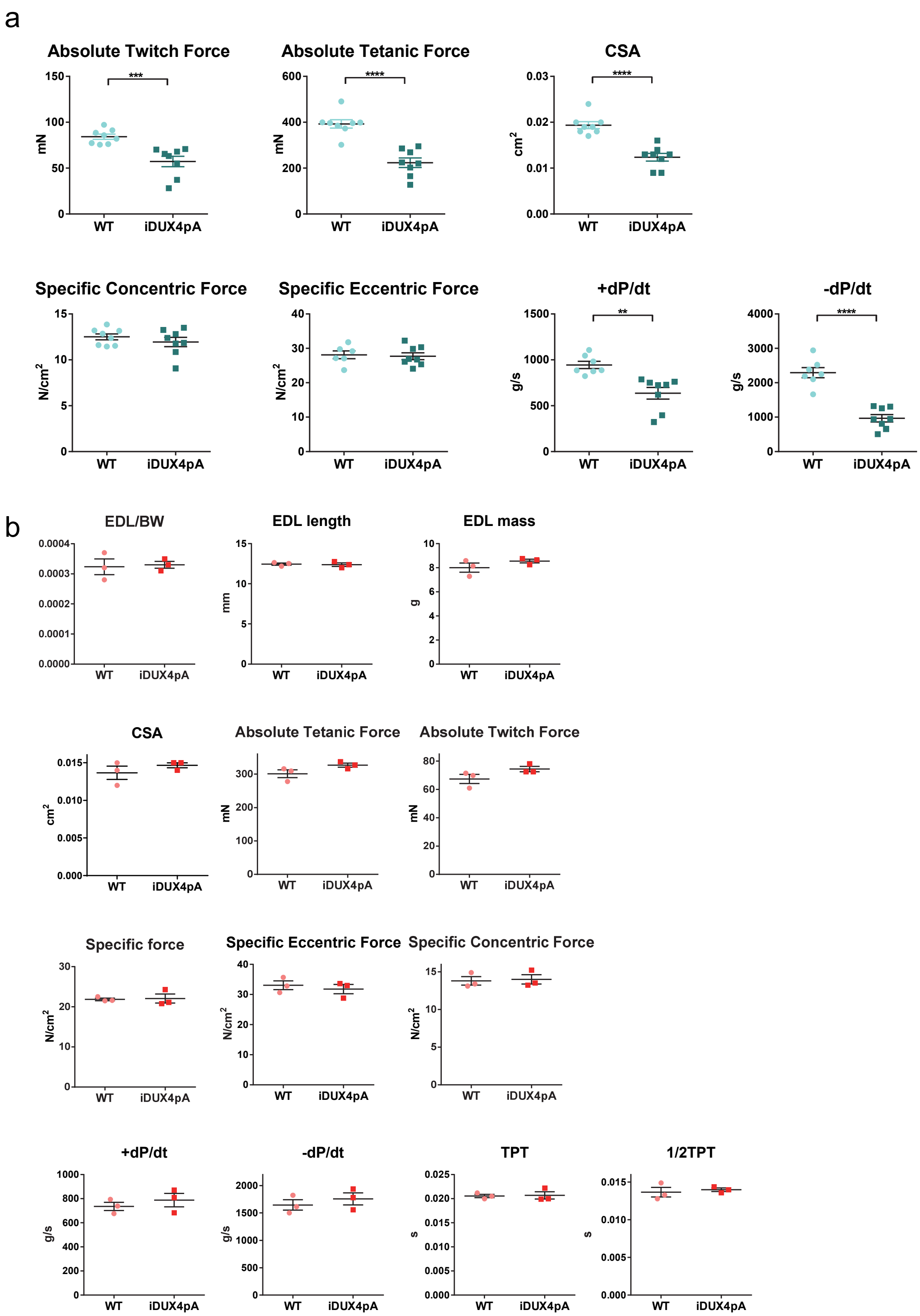

Supplementary Figure 2. EDL contractile force measurements in iDUX4pA males and females

(a) EDL cross sectional area (CSA), absolute twitch force, absolute isometric tetanic force, specific concentric force, specific eccentric force, rate of tetanic force generation (+dT/dt), rate of relaxation during tetanic contraction (–dP/dT) of 6 to 10 week old iDUX4pA males and WT siblings (n=8). (b) EDL force measurements in 6 week old iDUX4pA females and WT siblings (n=3). Rate of tetanic force generation (+dT/dt), rate of relaxation during tetanic contraction (–dP/dT), time to reach peak twitch force (TPT) and time to reach ½ relaxation during twitch (½ TPT). Data are presented as mean +/- SEM; T-test: *p<0.05, **p<0.01, ***p<0.001, ****p<0.0001. Note that all of the analyzed parameters in iDUX4pA females are similar to the WT controls.

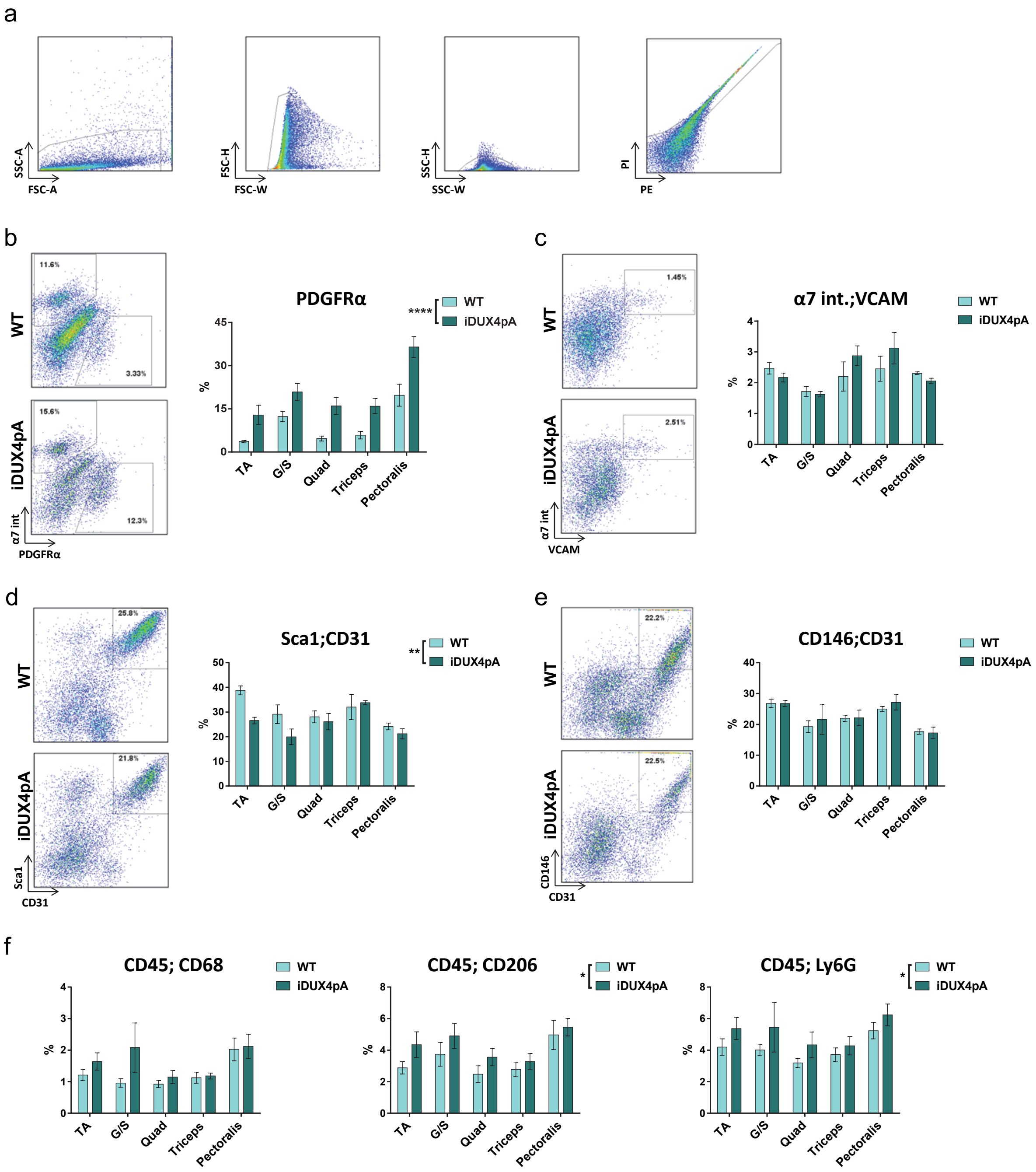

Supplementary Figure 3. FACS analysis of FAPs, myogenic progenitors, endothelial and inflammatory cells.

(a) Gating strategy for FACS analysis. Gates were defined based on unstained and single stained samples. Cells were gated on FSC and SSC, and dead cells were excluded by PI staining. (b) Representative FACS for Lin^{neg} (CD45^{neg}; CD31^{neg}) PDGFRα^{+} cells and percent of PDGFRα^{+} cells in various muscles. (c) Representative FACS for Lin^{neg} (CD45^{neg}; CD31^{neg}) integrin α7^{+} VCAM1^{+} cells, and percent of myogenic progenitors in various muscles. (d) Representative FACS for CD45neg Sca1+ CD31+ cells, and percent of Sca1+ endothelial progenitors in various muscles. (e) Representative FACS for CD45neg CD146 + CD31+ cells, and percent of pericytes in various muscles. (f) FACS analyses for CD45+ CD68+, CD45+ CD206+ and CD45+ Ly6G+ cells in various muscles. Samples from quadriceps are shown for representative FACS analyses. iDUX4pA and wild type males are 5 week old (n=4). Data are presented as mean +/- SEM; **p<0.01, ****p<0.0001 by two-way ANOVA with Sidak's post hoc test.

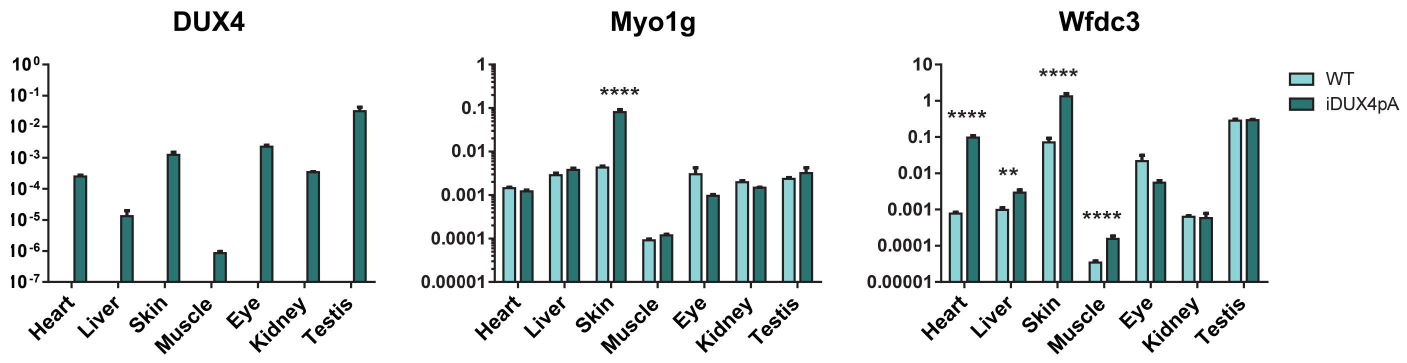

Supplementary Figure 4. Basal expression of DUX4 and target genes in various tissues.

RTqPCR for *DUX4*, *Myo1g* and *Wfdc3* in different tissues from iDUX4pA and wild type 9 week old male mice. Expression levels were normalized to the levels of *GAPDH* (n=4). Data are presented as mean +/- SEM; **p<0.01, ****p<0.0001 by two-way ANOVA with Sidak's post hoc test.

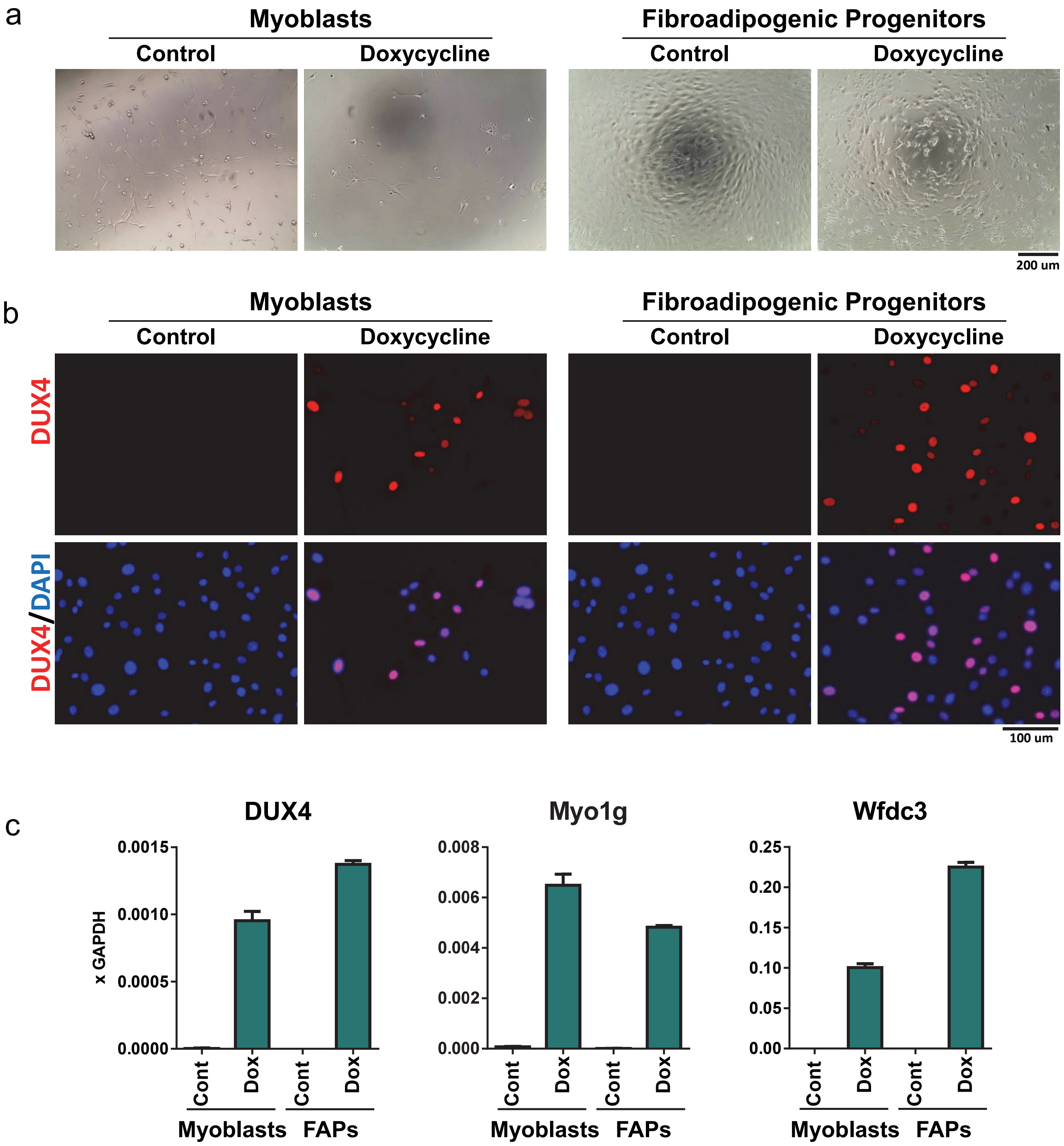

Supplementary Figure 5. DUX4 induces cell death in primary myogenic and fibroadipogenic progenitors.

(*a*) Cell morphology of myogenic (integrin α7+) and fibroadipogenic (PDGFRα+) progenitors isolated from muscle of iDUX4pA;ROSA-rtTA males cultured for 36 hours with 500 ng/mL doxycycline. (b) Immunostaining for DUX4 in primary cultures of sorted muscle integrin α7+ and PDGFRα+ cells induced with 500 ng/mL doxycycline for 12 hours. DAPI was used for nuclear staining. (c) RTqPCR analyses for DUX4 and DUX4 target genes in primary cultures of sorted muscle integrin α7+ and PDGFRα+ cells induced with 500 ng/mL doxycycline for 12 hours. Results are presented as fold difference to GAPDH (n=4, T-test: $p<0.0001$).

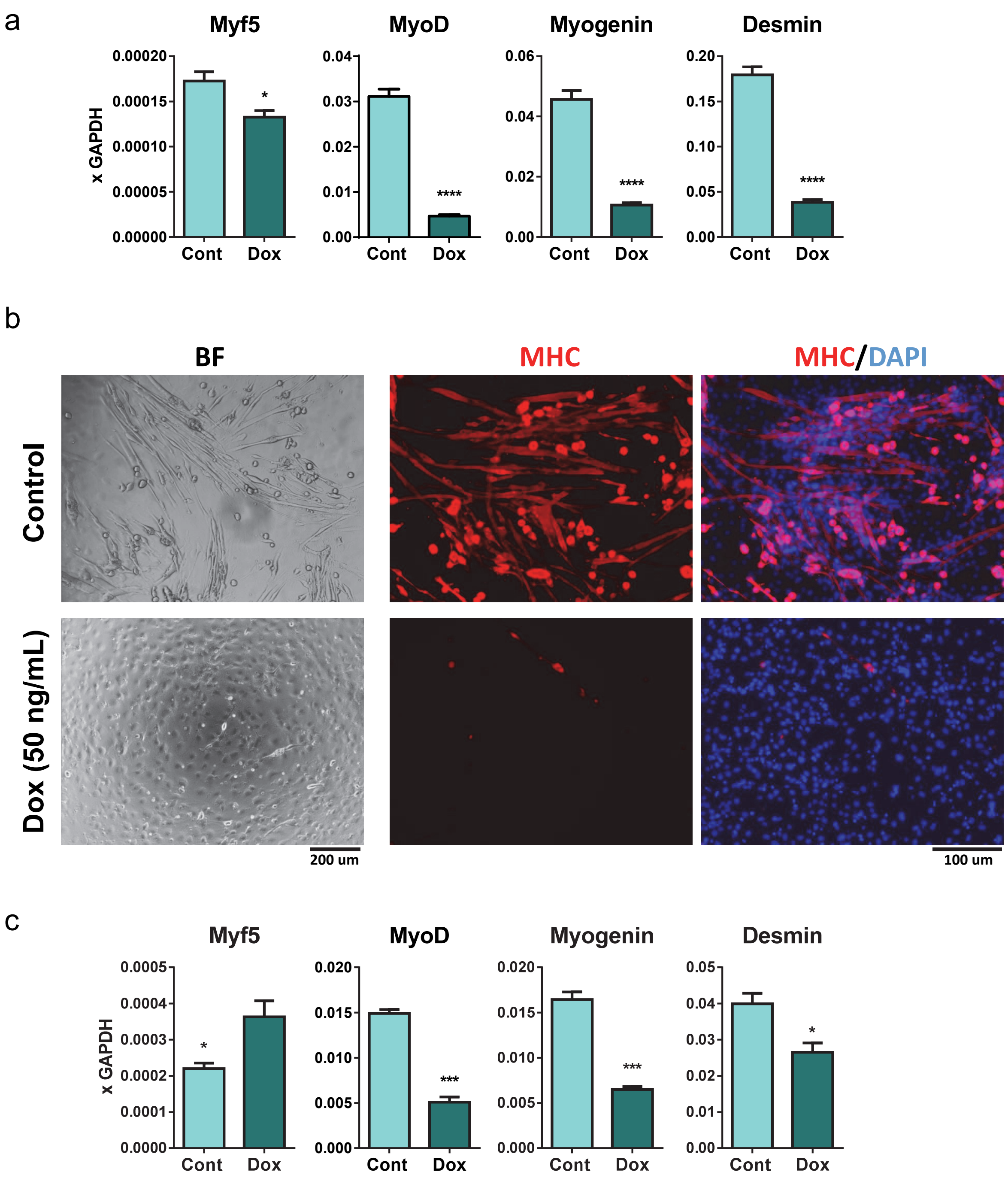

Supplementary Figure 6. Low levels of DUX4 expression inhibit primary myoblast differentiation.

(a) RTqPCR analyses for myogenic regulators in DUX4 proliferating integrin α7+ cells induced with 500 ng/mL doxycycline for 12 hours. Results are presented as fold difference to GAPDH (n=4). (b) Bright field (BF) image and immunostaining for MHC (myosin heavy chain (MF20 antibody)) of integrin α7+ at day 4 of myogenic differentiation. DUX4 was induced during 4 days of differentiation with 50 ng/mL doxycycline. DAPI was used for nuclear staining. (c) RTqPCR for myogenic genes in differentiated primary myogenic cells after 4 days of doxycycline induction (50 ng/mL). Results presented as fold difference compared to GAPDH (n=3). Data are presented as mean +/- SEM; T-test: *p<0.05, **p<0.01, ***p<0.001, ****p<0.0001.

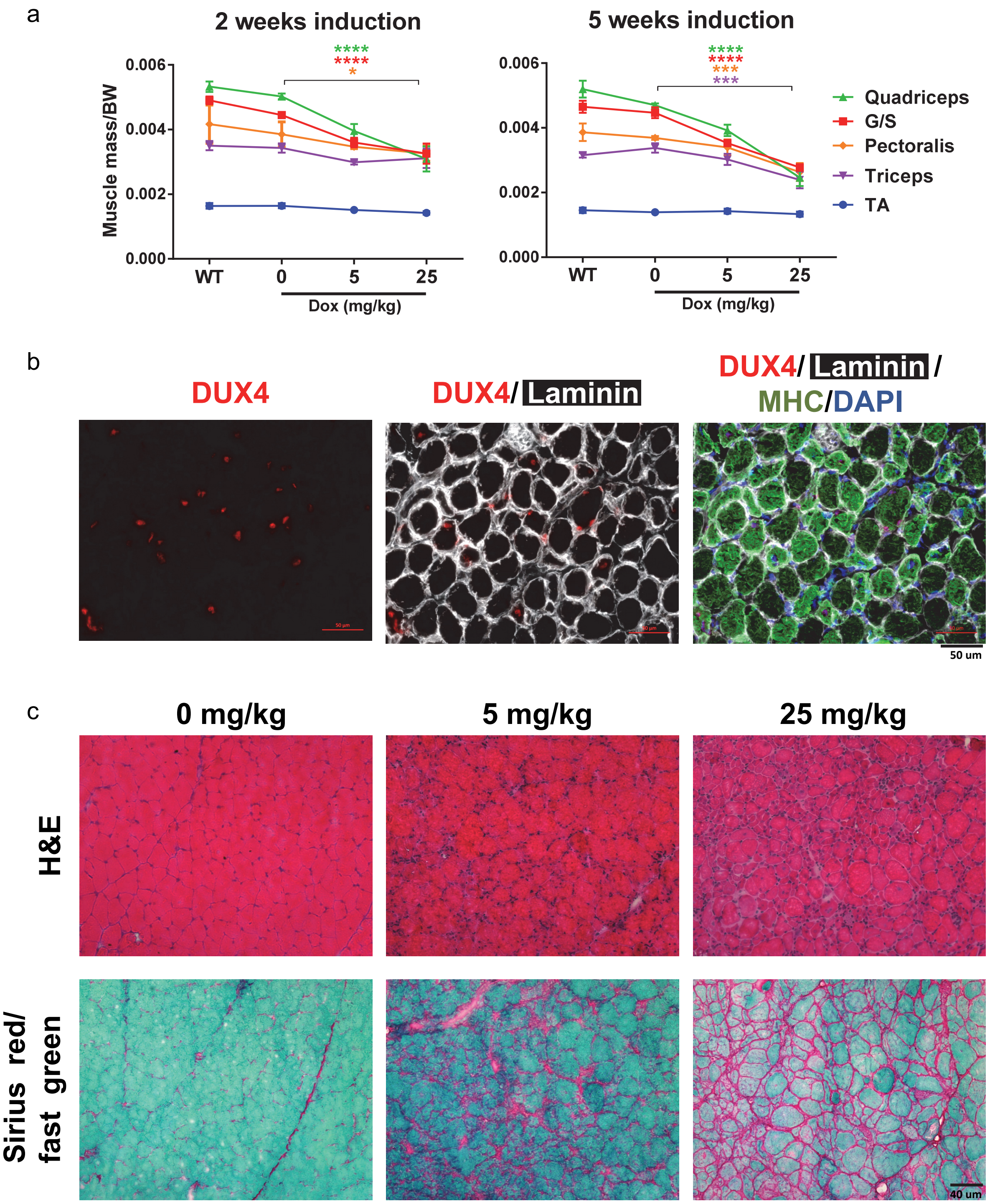

Supplementary Figure 7. DUX4 expression causes atrophy of various muscles in female mice.

(a) Mass of various muscles normalized to the body weight (BW) of iDUX4pA;HSA-rtTA females and WT siblings treated with doxycycline at indicated doses for indicated times (n=3). (b) Immunostaining for DUX4, MHC and Laminin in TA muscles of mice induced with 25 mg/kg doxycycline for 2 weeks. DUX4+ nuclei are within the Laminin-bounded domains (i.e. not interstitial) and within MHC+ cytoplasm (i.e. within muscle cells). (c) Hematoxylin and eosin (H&E), and Sirius red / fast green staining of sections from quadriceps of iDUX4pA;HSA-rtTA females induced with 5 and 25 mg/kg doxycycline for 5 weeks. Data are presented as mean +/- SEM; *p<0.05, **p<0.01, ***p<0.001, ****p<0.0001 by two-way ANOVA with Tukey's post hoc test.

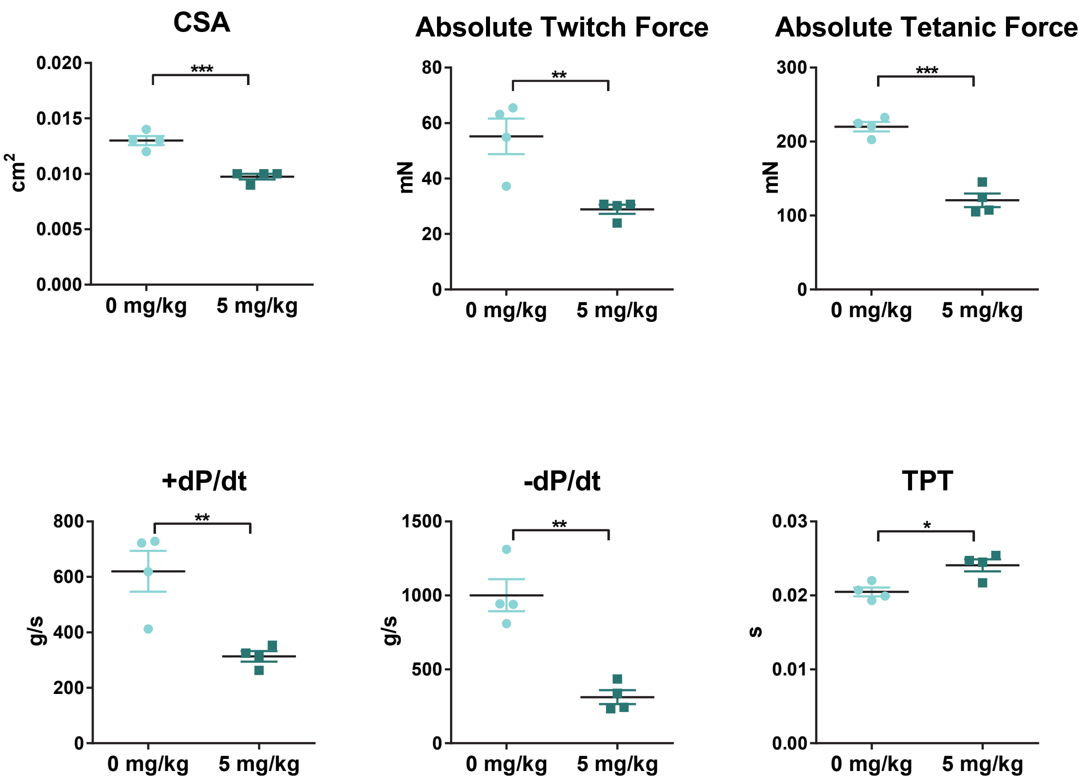

Supplementary Figure 8. Reduced EDL contractile force in induced males.

Various force parameters for EDL muscles from 10 week old iDUX4pA;HSA-rtTA males induced with doxycycline (5 mg/kg) for 28 days. (n=4, T-test: **p<0.01, ***p<0.001). Error bars indicate SEM.

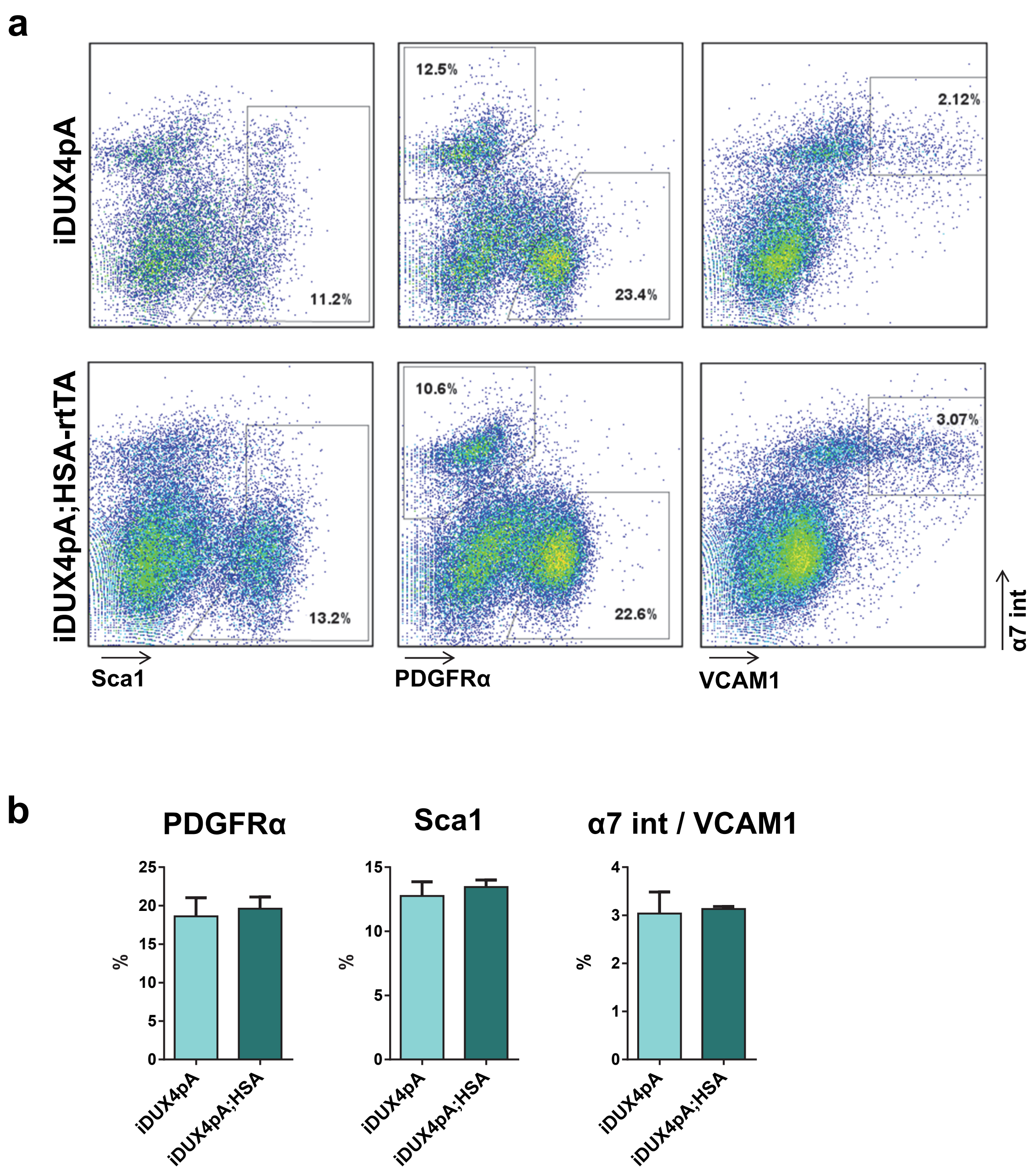

Supplementary Figure 9. FACS analysis of myogenic progenitors and FAPs in induced males.

(a) Representative FACS for Linneg (CD45neg; CD31neg) and PDGFRα+, Sca1+, or integrin α7+ / VCAM1+ positive cells in muscle from iDUX4pA;HSA-rtTA male mice induced with 25 mg/kg doxycycline for 14 days. (b) Percent of PDGFRα+, Sca1+ and integrin α7+ / VCAM1+ cells in muscle digests from pooled TA, gastrocnemius, soleus, quadriceps, pectoralis and triceps (n=3).

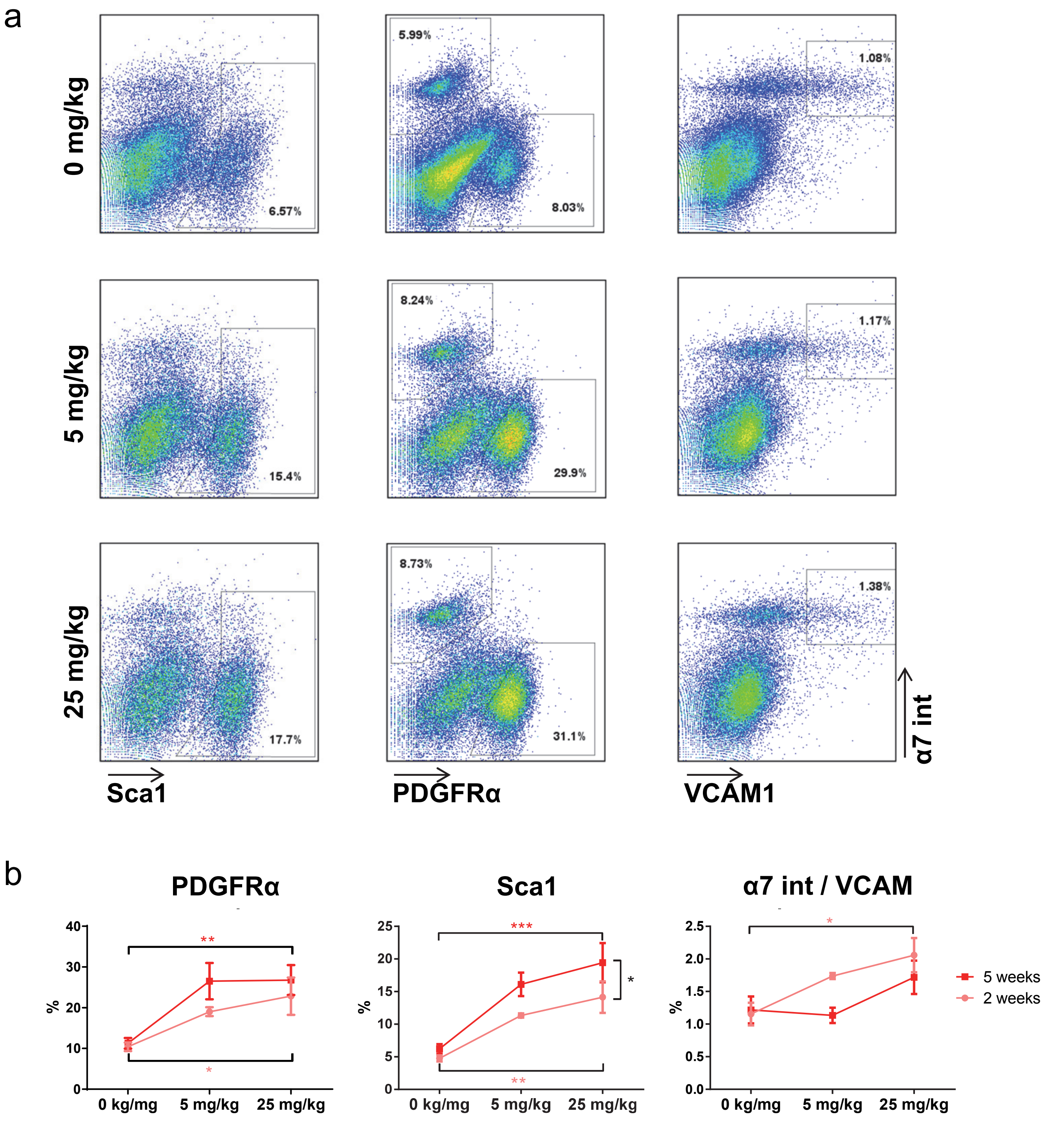

Supplementary Figure 10. Increased fibroadipogenic progenitors in induced iDUX4pA;HSA-rtTA females.

(a) Representative FACS for Lin-neg (CD45neg; CD31neg) and PDGFRα+, Sca1+, or integrin α7+ /VCAM1+ positive cells in muscle from iDUX4pA;HSA-rtTA females induced with 5 and 25 mg/kg doxycycline for 5 weeks. (b) Percent of PDGFRα+, Sca1+ and integrin α7+ / VCAM1+ cells in muscle digests from pooled TA, gastrocnemius, soleus, quadriceps, pectoralis and triceps (n=4). Data are presented as mean +/-SEM; *$p<0.05$, **$p<0.01$,***$p<0.001$, by two-way ANOVA with Tukey's post hoc test.

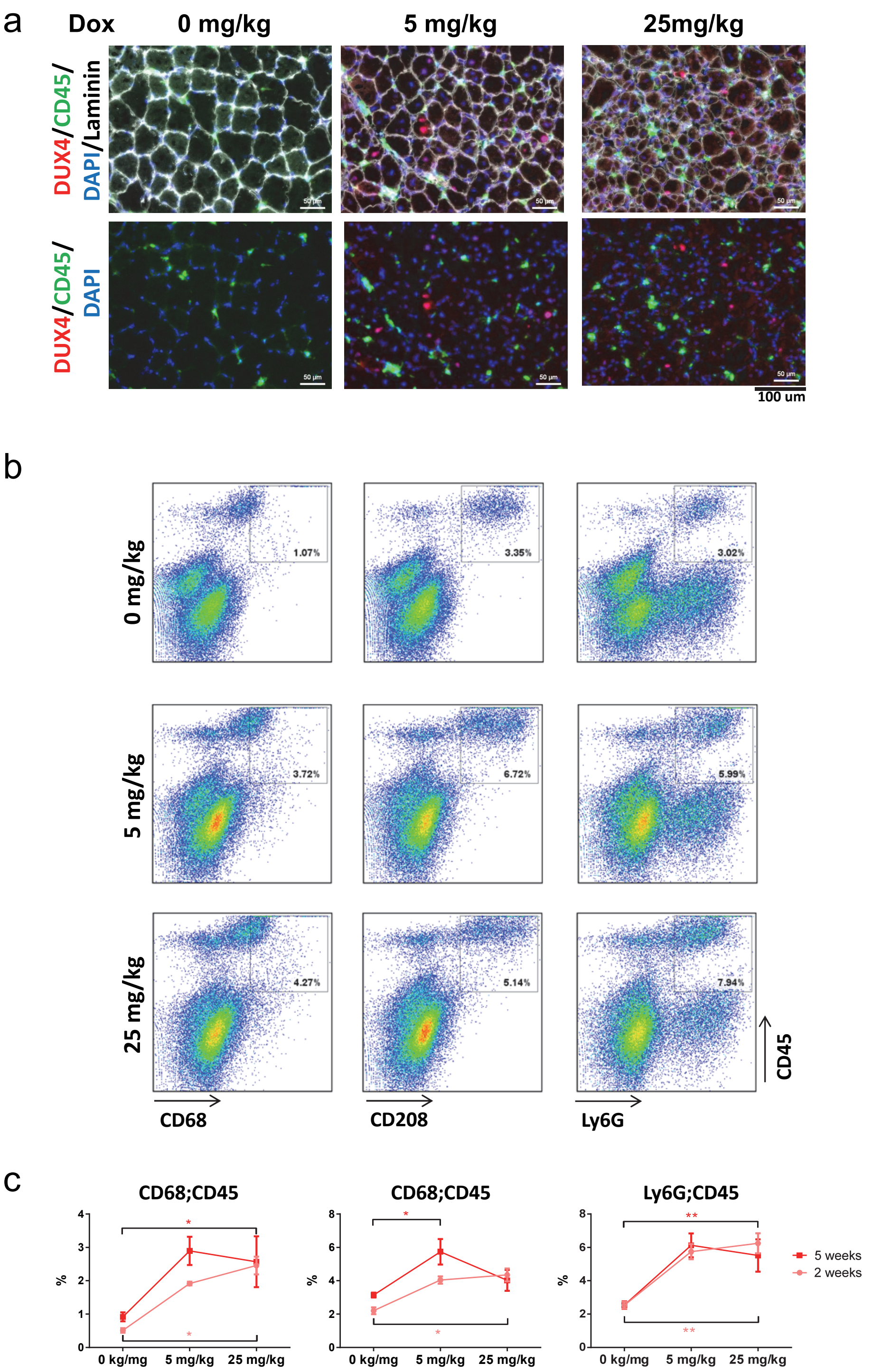

Supplementary Figure 11. Analysis of inflammatory infiltrate after DUX4 induction in females.

(a) Immunostaining for CD45, DUX4 and laminin in quadriceps from iDUX4pA;HSA-rtTA females induced with 25 mg/kg doxycycline for 14 days. DAPI was used for nuclear staining. (b) Representative FACS plots for CD45 vs. CD68, CD206 and Ly6G in females induced with 5 and 25 mg/kg dox for 5 weeks. (c) Summary of FACS analyses for CD45 and CD68, CD206+ or Ly6G+ in muscle of iDUX4pA;HAS-rtTA females induced with 5 and 25 mg/kg doxycycline for 2 and 5 weeks (n=4). Data are presented as mean +/- SEM; *p<0.05, **p<0.01 by two-way ANOVA with Tukey's post hoc test.

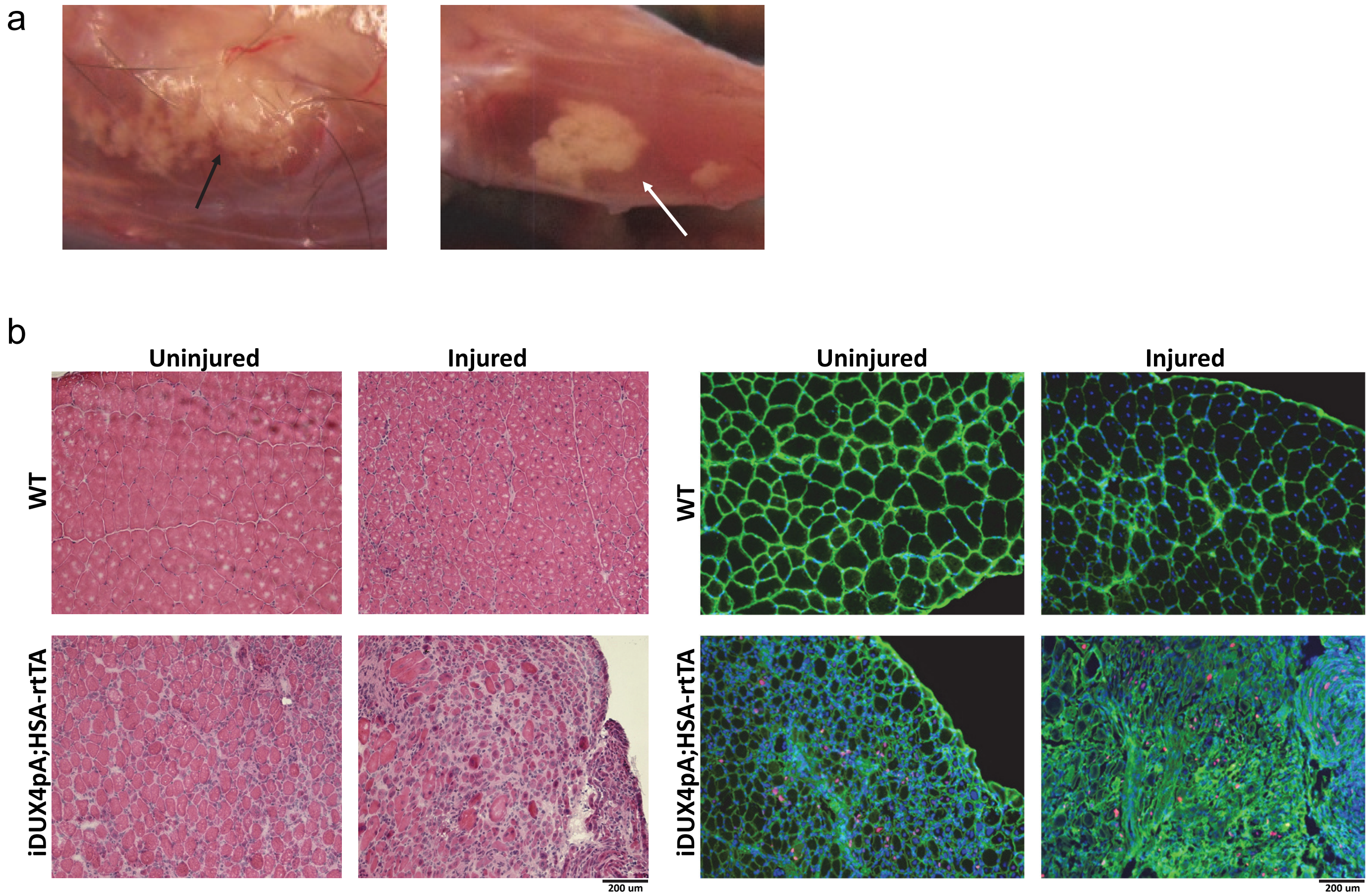

Supplementary Figure 12. Extensive atrophy and fibrosis at high dose induction.

(a) iDUX4pA;HSA-rtTA male mice 14 days post injury. Left TAs were injured with cardiotoxin and mice were induced with 100 mg/kg doxycycline. Black arrows indicate superficially visible regions of significant fibrosis in gluteal muscles; white arrow indicates zone of CTX-induced fibrosis in the injured TA muscle. (b) Hematoxylin and eosin (H&E) staining and immunostaining for DUX4, laminin and DAPI nuclear staining of uninjured and injured TA muscle sections.

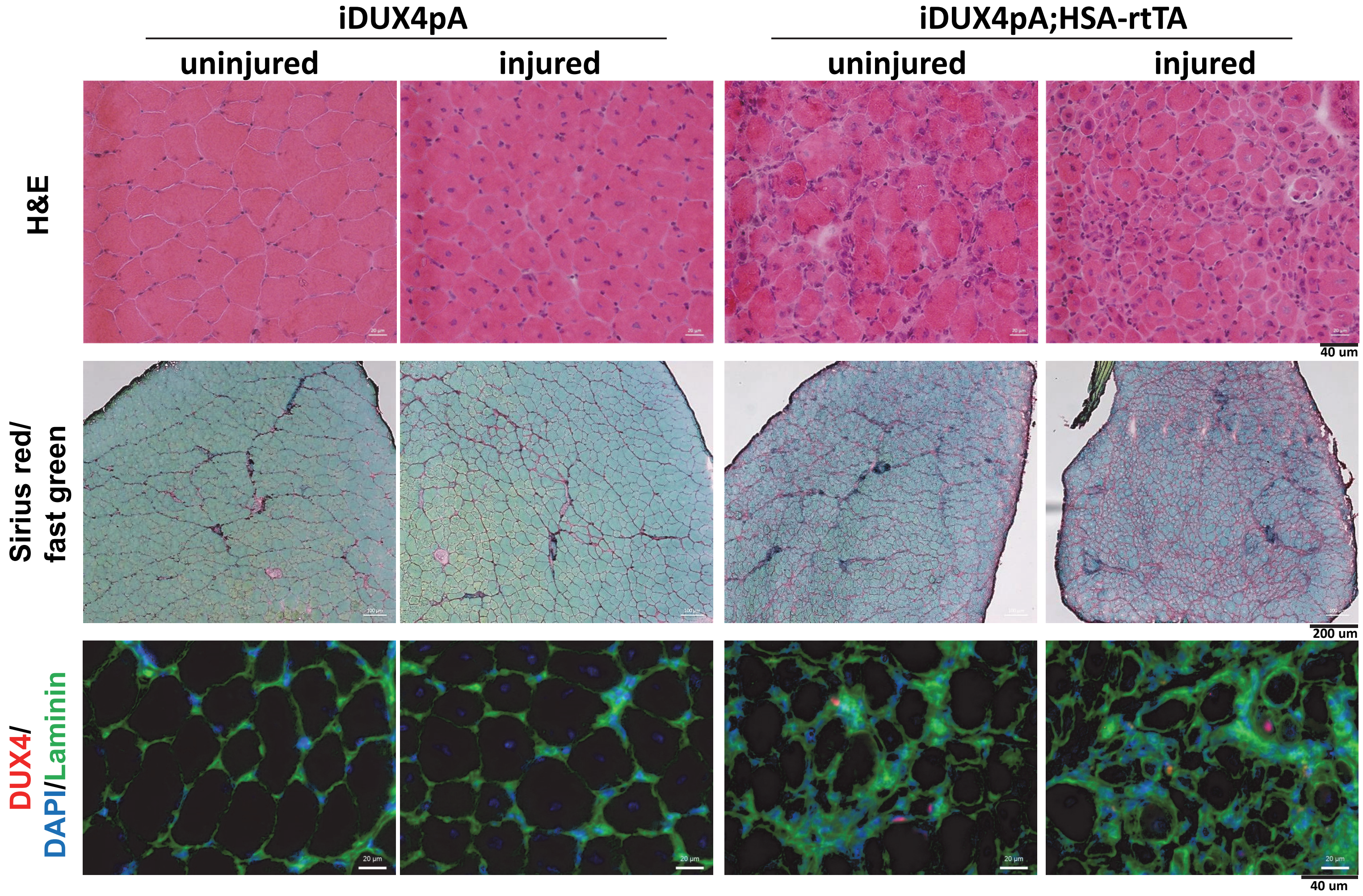

Supplementary Figure 13. Muscle-specific DUX4 impairs recovery after injury in female mice.

Hematoxylin and eosin (H&E) staining, Sirius red/fast green staining, and immunostaining for DUX4, laminin and DAPI of injured and uninjured TA muscle sections from iDUX4pA;HSA-rtTA female mice and littermate controls lacking the HSA-rtTA (thus not inducible for DUX4) 12 days post injury. Mice were induced with 25 mg/kg doxycycline for 12 days.

Gapdh	Mm99999915_g1
Myo1g	Mm00617991_m1
Wfdc3	Mm01243777_m1
PdgfRα	Mm00440685_g1
Bmp4	Mm00432087_m1
Myod1	Mm00440387_m1
Zscan4c	Mm02581232_m1
Azi2	Mm00479100_m1
Cdkn1a	Mm00432448_m1
Col1a1	Mm00801666_g1
Tgfβ1	Mm03024015_m1
Bmp4	Mm00432087_m1

Supplementary Table 1. Applied Biosystems RTqPCR probes used.

Name	Clone	Source		Application	Dilution
CD31	390	Rat Anti-Mouse	BD Pharmingen	FACS	1:200
CD106 (MVCAM.A)	429	Biotin Rat Anti-Mouse	BD Pharmingen	FACS	1:200
Alpha 7 integrin	R2F2	Rat	AbLab	FACS	1:200
CD104a (PDGFRα)	APA5	Rat Anti-Mouse	BD Pharmingen	FACS	1:200
Ly-6A/E (Sca-1)	D7	Rat Anti-Mouse	eBioscience	FACS	1:200
CD206 (MMR)	C068C2	Rat Anti-Mouse	bioLegend	FACS	1:200
CD68	FA-11	Rat Anti-Mouse	bioLegend	FACS	1:200
Ly-6G (Gr-1)	RB6-8C5	Rat Anti-Mouse	eBioscience	FACS	1:200
Cd11b	M1/70	Rat Anti-Mouse	eBioscience	FACS	1:200
CD146	ME-9F1	Rat Anti-Mouse	BD Pharmingen	FACS	1:200
Streptavidin FITC	11-4317-87	Rat	eBioscience	FACS	1:200
CD45	30-F11	Rat Anti-Mouse	BD Pharmingen	FACS, IF	1:200
IgG2b Iso Control	eB149/10H5	Rat	eBioscience	FACS	1:200
IgG2a Iso Control	eBR2a	Rat	eBioscience	FACS	1:200
DUX4	RD47c	Rabbit	R&D	IF	1:20
MHC	MF20	Mouse	Developmental Studies Hybridoma Bank	IF	1:20
Laminin	LAM-89	Mouse	Sigma	IF	1:1000
Laminin γ-1	3E10	Rat	Santa Cruz	IF	1:50
Alexa fluor 555	A21422	Goat Anti-Mouse	Invitrogen	IF	1:500
Alexa fluor 555	A21429	Goat Anti-Rabbit	Invitrogen	IF	1:500
Alexa fluor 488	A11029	Goat Anti-Mouse	Invitrogen	IF	1:500
Alexa fluor 488	A11034	Goat Anti-Rabbit	Invitrogen	IF	1:500
Alexa fluor 647	A21247	Goat Anti-Rat	Invitrogen	IF	1:500

Supplementary Table 2. Antibodies used
